# Plug-in Models: A Promising Direction for Molecular Generation

**DOI:** 10.34133/hds.0092

**Published:** 2023-10-09

**Authors:** Ningfeng Liu, Hongwei Jin, Liangren Zhang, Zhenming Liu

**Affiliations:** State Key Laboratory of Natural and Biomimetic Drugs, School of Pharmaceutical Sciences, Peking University, 100191 Beijing, P. R. China.

## Molecular Generation

The molecular generation has emerged as a powerful tool for computer-aided drug design in recent years, as it can explore a large and unknown chemical space and discover novel structures or scaffolds. Furthermore, a candidate compound needs to satisfy multiple criteria, such as target affinity, pharmacokinetics, toxicity, synthetic accessibility, etc., to pass clinical trials and meet industrial standards. Therefore, multi-objective methods have become a focal point of molecular generation and optimization. Several reviews have been published recently to summarize previous works in molecular generation and categorize them (Table). In this article, we propose a classification scheme based on both the model’s architecture and its practical use, namely, entrenched or plug-in models, especially for multi-objective molecular generation models. We argue that plug-in methods have superior flexibility in both model building and practical use, broader application potential, and higher performance boundaries, and they deserve more attention in the future.

**Table.** Examples of recent years’ reviews about molecular generation.

Authors	Year	Classification methods^a^
Bilodeau et al. [3]	2022	Representation, architecture, goal
Martinelli [4]	2022	Representation, architecture
Cheng et al. [5]	2021	Representation, architecture
Meyers et al. [6]	2021	Base
Mouchlis et al. [7]	2021	Architecture, goal, base
Xia et al. [8]	2019	Architecture
Xu et al. [9]	2019	Representation, architecture
Xue et al. [10]	2018	Architecture

^a^Representation—molecular representations, architecture—model architectures such as VAE or GAN, goal—goals to generate molecules, such as property or structure-constrained generation, and base—the fundamental idea such as atom-based, fragment-based, or reaction-based.

## Entrenched Models and Plug-in Models

In entrenched models of molecular generation, the model is fixed after being trained for a selected task. However, when facing new scenarios and needing to add previously unselected objectives, it is often necessary to redefine the training set and rebuild the model. Some examples of construction methods include building by inputting multi-objective information of molecules during training or generating molecules that meet specific distributions based on pre-trained models using transfer learning ideas. This type of distribution-based molecular generation should also be regarded as a multi-objective molecular generation because multi-objective information is already implied when selecting the training set. These entrenched models are limited in practical applications. For some teams that carry out multiple targets and disease projects at the same time, each project has different multi-objective requirements for molecules. They must use a large amount of labeled data to train multiple entrenched models for use and often find it difficult to use new data generated during the experiment. This becomes impossible without a cooperative computing team. In addition, in terms of the overall universality of the industry, entrenched models are difficult to promote as well-known platforms or software due to their narrow scope of use.

**Fig. 1.**
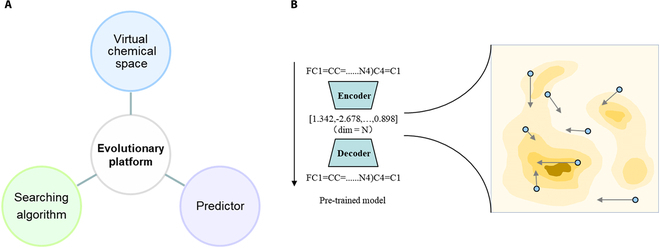
The concept of a promising plug-in molecular generation framework. (A) The components of the framework. (B) The virtual chemical space from the latent representation of a pair of encoder and decoder.

Plug-in multi-objective molecular generation models have higher flexibility. Their molecular generation algorithms and the part that implements multi-objectives are combined in a plug-in manner. Changing tasks and adding new objective plug-ins will not affect the molecular generation part and often do not require retraining the entire model. In this article, we propose that the virtual chemical space-search algorithm is a promising plug-in molecular generation framework (Fig. 1). It first constructs a pre-trained encoder–decoder model for molecules, mapping the vast chemical space to a virtual, continuous chemical space, and the encoded molecules can also be decoded back to their own structure. With the search algorithm, detectors can search for different multi-objectives in space to achieve multi-objective molecular generation. Some studies have successfully applied plug-in frameworks. In Winter et al.’s work [1], they used a pre-trained latent chemical space and searched for molecules in plug-in methods, and the QMO model developed by Hoffman et al. [2] uses the same chemical space for structural optimization.

Plug-in models have the potential to achieve higher applicability and performance than entrenched models and thus deserve more research attention. On the one hand, plug-in models can be used in various drug development scenarios without additional training, similar to docking programs or pharmacophore analysis. This lowers the barrier of use and facilitates dissemination. Moreover, for plug-in models based on virtual chemical space, any new quantifiable objective can be incorporated into the model as a new predictor plug-in, without affecting other molecular generation tasks that do not require the new objective. The same tool can continuously adapt to new demands, improve performance through updates, and even generalize to other domains such as materials science. Finally, in the framework based on virtual chemical space, the encoder–decoder and the search algorithm are both modular and pluggable, and can be updated flexibly. These plug-in features enable them to address problems and challenges [3–5] in molecular generations, such as data scarcity, goal practicality, synthetic accessibility, 3D information encoding, etc., in an independent and modular way. Furthermore, plug-in models can utilize other models in property prediction or target affinity prediction more easily, allowing for a gradual improvement of models through community collaboration.

## Future of Plug-in Molecular Generation Models

Plug-in models offer the advantage of flexibility in use and model updates. To fully exploit this potential, we suggest that future research should address the following aspects:

1. Developing a general plug-in predictor for target affinity. Ligand-based quantitative structure–activity relationship (QSAR) models can generate molecules with affinity for multiple targets, but they require a new training set for each target. This limits the flexibility of plug-in models and hinders their application to novel targets with no known ligands. However, target-based affinity prediction is challenging in the performance when generalizing to targets unseen to the model, which still needs efforts to improve.

2. Enhancing the multi-objective optimization capability of the search algorithm. A main challenge in building plug-in multi-objective models is the balance between the quality of generated molecules’ properties and the diversity of considered properties since the ultimate goal is to make molecules perfect and pass clinical trials. One benefit of distribution-based entrenched models is that they implicitly encode and generalize multiple objectives in the latent space, which ensures the quality of generation. For plug-in models that can switch objectives flexibly, it is essential to ensure that the search algorithm can effectively explore the available objectives and optimize them simultaneously.

3. Designing user-friendly tools with plug-in target predictor interfaces. The demands of drug development are diverse and dynamic. Some research groups may want to use their own data to build better prediction models, or some objectives may extend to underexplored areas such as phenotype-based drug discovery. Therefore, for users with some computational skills, a plug-in interface should be provided to enable them to easily integrate their own models into the tools for more customized molecular generation and to facilitate community collaboration in model updating.
